# Supplementary material for: Communicating Compositional Patterns
Source: Open Mind (Camb). 2020 Aug 1;4:25–39. doi: 10.1162/opmi_a_00032 (PMC8412198; doi:10.1162/opmi_a_00032)
Supplement: Supplementary file 1 [file opmi-04-25-s001.pdf]

# Communicating compositional patterns: Supporting Information

Eric Schulz

Department of Psychology

Harvard University

Francisco Quiroga

Department of Experimental Psychology

University College London

Samuel J. Gershman

Department of Psychology

Harvard University

Author Note

Correspondence concerning this project should be addressed to Eric Schulz, Harvard University, 52 Oxford Street, Room 295.08, Cambridge, MA 02138, E-mail: ericschulz@fas.harvard.edu.

## Communicating compositional patterns: Supporting Information

### Data, descriptions and analysis code

All data, analysis script and experimental code can be found online at:

`https://github.com/anonymous/function_communication`

All descriptions, originals and redrawn patterns can be found online at:

`https://anonymous.github.io/comcomppats.pdf`

### Non-compositional kernel

The non-compositional kernel is based on an approximation of the spectral density of function in a Gaussian Process framework. Letting $\tau = x - x' \in \mathbb{R}^P$, then

$$k(\tau) = \int_{\mathbb{R}^P} e^{2\pi i s^\top \tau} \psi(\mathrm{d}s). \tag{1}$$

If $\psi$ has a density $S(s)$, then $S$ is the spectral density of $k$; $S$ and $k$ are Fourier duals (Rasmussen & Williams, 2006). Thus, a spectral density over the kernel space fully defines the kernel. Furthermore, every stationary kernel can be expressed as a spectral density. Wilson and Adams (2013) showed that the spectral density can be approximated by a mixture of $Q$ Gaussians, such that

$$k(\tau) = \sum_{q=1}^{Q} w_q \prod_{p=1}^{P} \exp\left(-2\pi^2 \tau_p^2 \upsilon_q^p\right) \cos\left(2\pi \tau_p \mu_q^{(p)}\right), \tag{2}$$

where the $q$th component has mean vector $\mu_q = \left(\mu_q^{(1)}, \ldots, \mu_q^{(P)}\right)$ and a covariance matrix $\mathbf{M}_q = \mathrm{diag}\left(\upsilon_q^{(1)}, \ldots, \upsilon_q^{(P)}\right)$.

### Statistical tests

We report all statistics using both frequentist and Bayesian tests. Frequentist tests are presented alongside their effect sizes, i.e. Cohen's d (Cohen's d; Cohen, 1988). Bayesian statistics are expressed as Bayes factors (BFs). A Bayes factor quantifies the likelihood of the data under the alternative hypothesis $H_A$ compared to the likelihood of

the data under the null hypothesis $H_0$. For example, a $BF$ of 10 indicates that the data are 10 times more likely under $H_A$ than under $H_0$; a $BF$ of 0.1 indicates that the data are 10 times more likely under $H_0$ than under $H_A$. We use the "default" Bayesian $t$-test as proposed by Rouder and Morey (2012) for comparing independent groups, using a Jeffreys-Zellner-Siow prior with its scale set to $\sqrt{2}/2$. The Bayes factor for the correlation between the judges' ratings is based on Jeffrey's test for linear correlation as put forward by Ly, Verhagen, and Wagenmakers (2016). We approximate the Bayes factor between two different mixed-effects regressions by applying bridge sampling (Gronau et al., 2017).

## Validating the description ratings

We validated the two judges' ratings by running an additional experiment in which 20 participants (10 females, mean age=36.2, SD=14.7, fee=$2) on MTurk sequentially rated 50 randomly sampled descriptions while also seeing the matching pattern. Participants used the same rating scale as the two judges, ranging from 1 (bad description) to 5 (very good description). The resulting mean ratings and the judges' ratings correlated highly: $r(29) = 0.86$, $t = 8.66$, $p < .001$, $BF > 100$. Furthermore, to assess if the pre-selection had potentially biased our model comparison, we analyzed how well both models described all patterns before and after the selection. This showed no difference for either the compositional ($BF = 0.2$) or the non-compositional model ($BF = 0.2$). Moreover, both models described the selected patterns about equally well ($BF = 0.8$).

## Wavelet transform similarity measure

The discrete wavelet Haar transform performs a scale-wise decomposition of a pattern in such a way that most of the energy of the data can be represented by a few coefficients. The main idea behind this measure is to replace the original series by its wavelet approximation coefficients $\mathbf{a}$, and then to measure the dissimilarity between the wavelet approximations. We use the R-package `TSclust` (Montero, Vilar, et al., 2014) to find the appropriate scale of the transform. We then measured the dissimilarity

between two patterns $x_1$ and $x_2$ by the Euclidean distance at the selected scale: $d(x_1, x_2) = ||\mathbf{a}_1 - \mathbf{a}_2||$.

## Assessing other distance measures

We also compared compositional and non-compositional patterns using two other distance measure. The first one is the absolute distance of the actual points participants put onto the canvas and the closest points (on the x-axis) of the true patterns. This measure led to a smaller error for compositional than for non-compositional patterns ($t(49) = 3.38$, $p = .001$, $d = 0.48$, $BF = 20.9$). The second one is the absolute distance between two generalized additive models (Hastie, 2017), one fitted to participants' drawings and one to the true underlying pattern. In contrast to the smoothing lines used in the main text, this regression was not forced to go through every point, but rather to be a more compact representation of the drawn patterns. Using this distance measure, we found the same result as before, with a smaller error for compositional than non-compositional patterns ($t(49) = 2.72$, $p = .009$, $d = 0.38$, $BF = 4.1$). We therefore conclude that compositional patterns are more communicable than non-compositional patterns, independent of the distance measure.

## Lesioned model comparison

We assessed how well the compositional model captured the difficulty of communicating different patterns (as measured by the Wavelet distance) when lesioning the model by removing kernels from the grammar. This showed that all parts of the grammar were required for good performance, since the model described errors worse without the periodic kernel ($BF = 25.7$), without the Radial Basis Function kernel ($BF > 100$) and without the linear kernel ($BF = 48.2$).

## Comparing against other smoothness-based models

We also compared the compositional kernel against multiple smoothness-only models in terms of how they captured participants' performance. We used the Matérn class of kernel functions to encode the underlying smoothness of a function. The

Matérn covariance between two points separated by $\tau$ distance units is

$$C_\nu(\tau) = \sigma^2 \frac{2^{1-\nu}}{\Gamma(\nu)} \left(\sqrt{2\nu}\frac{\tau}{\rho}\right) K_\nu \left(\sqrt{2\nu}\frac{\tau}{\rho}\right) \tag{3}$$

where $\Gamma$ is the gamma function, $K_v$ is the modified Bessel function of the second kind, and $\rho$ and $\nu$ are non-negative covariance parameters. For $k_{1/2}$ the Matérn kernel is the Ornstein-Uhlenbeck kernel, which is a highly unsmooth model of functional patterns. As $k$ increases, the Matérn kernel expects smoother functions. For $k \to \infty$ the Matérn kernel is equivalent to a Radial Basis function kernel. We compare Matérn kernels with $k = [0.5, 1.5, 2.5, \infty]$ to the compositional model based on how well they predict participants' ability to communicate patterns, measured by the wavelet distance. The compositional model predicted the communicability of patterns better than any of the smoothness-based models with all Bayes factors being larger than 100.

# References

Cohen, J. (1988). *Statistical power analysis for the behavioral sciences. 2nd.* Hillsdale, NJ: erlbaum.

Gronau, Q. F., Sarafoglou, A., Matzke, D., Ly, A., Boehm, U., Marsman, M., ... Steingroever, H. (2017). A tutorial on bridge sampling. *Journal of mathematical psychology*, *81*, 80–97.

Hastie, T. J. (2017). Generalized additive models. In *Statistical models in s* (pp. 249–307). Routledge.

Ly, A., Verhagen, J., & Wagenmakers, E.-J. (2016). Harold Jeffreys's default Bayes factor hypothesis tests: Explanation, extension, and application in psychology. *Journal of Mathematical Psychology*, *72*, 19–32.

Montero, P., Vilar, J. A., et al. (2014). Tsclust: An r package for time series clustering. *Journal of Statistical Software*.

Rasmussen, C., & Williams, C. (2006). *Gaussian processes for machine learning.* MIT Press.

Rouder, J. N., & Morey, R. D. (2012). Default Bayes Factors for Model Selection in Regression. , *47*, 877–903. doi: 10.1080/00273171.2012.734737

Wilson, A. G., & Adams, R. P. (2013). Gaussian process kernels for pattern discovery and extrapolation. *arXiv preprint arXiv:1302.4245*.
